# The Emotional Intelligence of Japanese Mental Health Nurses

**DOI:** 10.3389/fpsyg.2019.02004

**Published:** 2019-08-30

**Authors:** Shinichiro Ishii, Etsuo Horikawa

**Affiliations:** ^1^School of Nursing, Jichi Medical University, Shimotsuke, Japan; ^2^Research and Education Center for Comprehensive Community Medicine, Faculty of Medicine, Saga University, Saga, Japan

**Keywords:** awareness, emotional intelligence, Japan, mental health, nurses

## Abstract

The aim of this study was to measure emotional intelligence in nurses in Japan, and to elucidate the characteristics of mental health nurses. Anonymous, self-administered questionnaires were distributed to 206 nurses working in three psychiatric hospitals and two internal medicine hospitals in Japan. The number of participants included in the analysis was 159 (valid response rate, 77.2%), of which 87 were mental health nurses. Emotional intelligence was measured using the Japanese version of the Wong and Law Emotional Intelligence Scale. In the analysis, the emotional intelligence of mental health nurses and internal medicine nurses was compared using a *t*-test. The results of a *t*-test comparing emotional intelligence scale scores between the mental health nurses and internal medicine nurses showed that the total mean score and Self-Emotions Appraisal score were significantly lower in mental health nurses than in internal medicine nurses. The self-awareness of mental health nurses was significantly lower than that of nurses in other fields (*P* < 0.001, Cohen’s *d*: 0.65). The results of this study provide a basis for future research on the emotional intelligence of nurses in Japan. Development of an emotional intelligence scale based on the characteristics of Japanese nurses will be important regarding nurses’ assessment of their own emotions and the emotions of others. The kinds of people nurses are involved with and the content of their support for others also need to be clarified.

## Introduction

The ability of nurses to make a wide range of responses and choices in various situations and contexts, in other words, clinical judgment, is a high-level practical nursing skills (Tanner, 2006; Benner et al., 2008; Maeda, 2012; Lusk and Fater, 2013). In particular, mental health nurses (MHNs) determine their nursing actions by first noticing things that are unusual or different from before and developing a sense of discomfort in the form of worry or unease, and following up by detecting violence and infliction of harm to self or others, selecting the responding staff nurse based on depth of involvement or relationship with the patient in question, and responding flexibly depending on the situation or context. They then need to analyze the patient responses and their own speech and behavior while these nursing actions are being performed or by self-reflection after the actions.

Reflective processes have been described as being “triggered by an awareness of uncomfortable feelings and thoughts. This arises from a realization that, in a situation, the knowledge one was applying was not sufficient in itself to explain what was happening in that unique situation” (Atkins and Murphy, 1993). Like observation, it requires sufficient training (Baly, 1997). However, reflection is not limited to competent or proficient nurses; it is also seen in nurses with little practical psychiatric clinical experience. Psychiatric clinical practice demands greater ability for reflection from nurses themselves, which is referred to as “therapeutic use of self” (Warelow and Edward, 2007; Chaffey et al., 2012; Felton et al., 2017). The basic, foundational skill for reflection is self-awareness, which is possible by assessment and analysis of one’s own emotions (Fitzpatrick, 2006; Harrison and Fopma-Loy, 2010; Atkins and Schutz, 2013). Therefore, taking reflection to be a reconstruction of experience, we focused on emotions that are strongly perceived as subjective experience. Our emotions can be assessed with an emotional intelligence (EI) scale.

Emotional intelligence is the ability of individuals to deal with emotions (Salovey and Mayer, 1990), and consists of four component abilities: (1) Self-Emotions Appraisal (SEA), (2) Others-Emotions Appraisal (OEA), (3) Use of Emotion (UOE), and (4) Regulation of Emotion (ROE) (Wong and Law, 2002; Law et al., 2004; Toyota et al., 2007). Previous studies on EI include cross-sectional studies of student subjects (Chan and Hamamura, 2016; Orak et al., 2016; Stiglic et al., 2018), investigations of the relationship with burnout (Taylor et al., 2015; Chao et al., 2016), and investigations of the relationship with nursing management (Lucas et al., 2008; Spano-Szekely et al., 2016). Actual EI in nursing outside Japan has been demonstrated in countries including Netherlands, South Korea, and China, and while the characteristics differ depending on the assessment scale used and the country surveyed, the need for continuous education and follow-up has been suggested (Van Dusseldorp et al., 2011; Wang et al., 2014, 2018; Sims, 2017). However, EI in Japanese nurses has yet to be elucidated.

Ishii (2018) surveyed EI of 431 MHNs in Japan. The results showed a significant positive correlation between EI and age, but no correlation was found between EI and nursing experience. The reason is that about 60% of MHNs are 40 years or older, comparatively older than nurses in other fields, and the proportion of licensed practical nurses is higher in older age groups. Moreover, this survey targeted only MHNs and the characteristics of MHNs compared with other nursing fields have not been clarified. Therefore, there is a need to clarify the EI that MHNs need to nurture reflective practice and self-awareness in psychiatric clinical practice. Based on specialized knowledge and skills in mental health nursing, length of experience in clinical nursing practice, and literature review (Takei, 2001; Ishii et al., 2015; Itabashi et al., 2016; Ishii, 2018), we predicted that EI of MHNs is at a higher level than nurses in other fields.

The purpose of this study was to measure the EI of Japanese nurses and among them to clarify the characteristics of MHNs.

## Materials and Methods

### Participants and Procedures

The participants of this study were Japanese nurses in their 20s and 30s. Anonymous, self-administered questionnaires were distributed to 206 nurses working in three psychiatric hospitals and two internal medicine hospitals in the Kyushu region from December 2014 to March 2015.

We provided a written explanation of the purpose and methods of the study to participants in advance, including that return of the questionnaire sheet would be taken as consenting to participate in the study, and that individuals would incur no disadvantage whatsoever by refusing to participate. The anonymity of individuals and the protection of privacy were guaranteed by coding the questionnaires after each of the consenting participants completed the questionnaire and sealed it in an envelope. Questionnaires with incomplete responses were excluded, leaving 159 participants (valid response rate, 77.2%) included in the final analysis.

### Measures

The questionnaire included participants’ age, sex, qualifications, number of years of nursing experience, and an EI scale. For EI, we used the Japanese version (Toyota and Yamamoto, 2011) of the Wong and Law Emotional Intelligence Scale (WLEIS) developed by Wong and Law (2002).

Wong and Law (2002) developed the WLEIS, and evaluated its reliability and validity. Internal consistency reliability for the four factors abilities ranged from 0.83 to 0.90. The WLEIS has been used in surveys of the EI of nursing students, medical students, nurses, and doctors (Weng, 2008; Weng et al., 2011; Ishii et al., 2015; Kim and Han, 2015; Sim and Bang, 2015). The WLEIS (J-WLEIS) is composed of 16 items in the following four factors:

(1) (SEA) Appraisal and expression of emotion in the self: This subscale assesses the individual’s ability to understand and express their deep emotions (sample item: “I have good understanding of my own emotions”).
(2) (OEA) Appraisal and recognition of emotion in others: This subscale reflects the individual’s ability to perceive and understand the emotions of individuals around them (sample item: “I have good understanding of the emotions of people around me”).
(3) (UOE) Use of emotion to facilitate performances: This subscale echoes the individual’s ability to utilize and direct his/her emotions toward constructive activities and personal performances (sample item: “I always tell myself I am a competent person”).
(4) (ROE) Regulation of emotion in the self: This subscale evaluates the individual’s ability to regulate his/her emotions, which facilitates rapid and successful recovery after psychological distress (sample item: “I have good control of my own emotions”).

The response format of each item is a 7-point Likert scale, with a score range of 1–7 points. Higher scores indicate higher EI.

### Statistical Analysis

The mean score and standard deviation of each factor (SEA, OEA, UOE, and ROE) on the J-WLEIS were calculated. The participants were divided into two groups, one comprised of MHNs and another comprised of internal medicine nurses (IMNs), and mean scores were compared using an unpaired *t*-test. Moreover, a chi squared test was used to compare the distributions of MHNs and IMNs.

Additionally, the effect size (Cohen’s *d*) was calculated for the score between the two groups. The effect size for Cohen’s *d* was taken as small if < 0.2, medium if between 0.2 and 0.5, moderate if between 0.5 and 0.8 and large if > 0.8 (Cohen, 1988).

Cronbach’s alpha was calculated for the items of each factor to examine internal consistency. The significance level was set at 5%. SPSS 25.0 for Windows (SPSS, Chicago, IL, United States) was used for all statistical analyses.

## Results

### Participants Demographics

We confirmed the basic attributes of the 159 participants. The participants included 87 MHNs and 72 IMNs. By sex, there were 108 women and 51 men. Men made up only about 7% of IMNs, but more than half of all MHNs (χ*^2^* = 38.14, *P* < 0.001). More than 70% of the participants were registered nurses. The mean age was 29.7 ± 5.7 years and the mean years of nursing experience was 6.3 ± 4.9. Participant demographics are shown in Table 1.

**TABLE 1:** Participants demographics data by group.

		**Total**		**MHNs (*n* = 87)**		**IMNs (*n* = 72)**		χ^2^
		***N***	**%**	***n***	**%**	***n***	**%**	
Sex								38.14^∗∗∗^
	Female	108	67.9	41	47.1	67	93.1	
	Male	51	32.1	46	52.9	5	6.9	
License								0.28
	RN	116	73.0	62	71.3	54	75.0	
	LPN	43	27.0	25	28.7	18	25.0	
		Mean (SD)		Mean (SD)		Mean (SD)		
Age (years)		29.7 (5.7)		31.2 (5.4)		27.9 (5.6)		27.61
Total years of nursing experience		6.3 (4.9)		6.7 (4.8)		5.8 (4.9)		28.14

**N* = 159, Chi-squared test ^∗∗∗^*P* < 0.001. IMNs, internal medicine nurses; LPN, licensed practical nurse; MHNs, mental health nurses; RN, registered nurse; SD, standard deviation.*

### EI

Internal consistency was determined by calculating Cronbach’s alpha coefficient. For the whole J-WLEIS this was 0.85, while for SEA it was 0.84, for OEA it was 0.72, for UOE it was 0.70, and for ROE it was 0.83. These scores supported the reliability of the J-WLEIS (Nunnally, 1978).

A comparison of J-WLEIS scores between the two groups is shown in Table 2. The participants’ J-WLEIS showed the highest scores for SEA (4.94 ± 0.84) and the lowest scores for UOE (4.00 ± 0.72). SEA and UOE showed the same trends in both groups.

**TABLE 2:** J-WLEIS scores.

	**Total**		**MHNs (*n* = 87)**		**IMNs (*n* = 72)**		***t*-value**	**d.f.**	***P*-value**	**Cohen’s *d***
	**Mean**	**SD**	**Mean**	**SD**	**Mean**	**SD**				
Total	4.44	0.56	4.35	0.56	4.54	0.54	−2.244	156	0.026^∗^	0.35
SEA	4.94	0.84	4.70	0.81	5.22	0.80	−4.048	156	0.000^∗∗∗^	0.65
OEA	4.71	0.60	4.63	0.55	4.81	0.64	−1.910	156	0.058	0.30
UOE	4.00	0.72	3.96	0.72	4.06	0.73	−0.830	156	0.408	0.14
ROE	4.10	0.90	4.08	0.85	4.09	0.96	0.035	156	0.972	0.01

**N* = 159, *t*-test (two-tailed). ^∗^*P* < 0.05, ^∗∗∗^*P* < 0.001. IMNs, internal medicine nurses; MHNs, mental health nurses; OEA, others-emotions appraisal; ROE, regulation of emotion; SD, standard deviation; SEA, self-emotions appraisal; UOE, use of emotion.*

The total mean score and mean scores for the four factors on the J-WLEIS were compared between the two groups using a *t*-test. The results showed that the MHNs had a significantly lower SEA score than the IMNs (*P* < 0.001). Cohen’s *d* of 0.65 has shown that the effect size is moderate.

## Discussion

Accurate clinical judgments are high-level practical nursing skills. Assessment and analysis of one’s own emotions are important in order to raise “self-awareness,” the skill that provides the foundation for reflection related to clinical judgments. Therefore, we decided to compare and clarify the actual EI of MHNs compared with nurses in non-psychiatric clinical practice.

Considering the specialized knowledge and skills in psychiatric nursing, the length of experience in clinical nursing practice, and a review of the literature in Japan, we expected that the EI of MHNs would be at a higher level than that of nurses in other fields. However, our results showed that the EI of MHNs was lower than that of IMNs in all scores on the J-WLEIS. In particular, SEA, which is related to self-awareness, was statistically significantly lower in MHNs than in IMNs.

Among studies on EI, this study had results opposite to those of Sims (2017) and Van Dusseldorp et al. (2011), who focused on MHNs. Three reasons may be considered for this. First is that the scales used were different. Second is that MHNs interact with people with mental disorders and their families, and therefore may overprepare themselves because of a feeling that they “have to understand the feelings of others,” or “should feel empathy with others,” and so may abandon or try to ignore their own feelings (Cunico et al., 2012). Third is that while psychiatric treatment in Japan is currently being shifted to community medicine, there are still many patients under long-term hospitalization (Tachimori et al., 2015; Shinjo et al., 2017). In such a treatment environment, there is a strong possibility of an effect from excessive closeness in the relationships built up between patients and nurses and the length of those relationships.

Nursing is said to be emotional labor (Hochschild, 2012; Smith, 2012). Itabashi et al. (2016), while stating that MHNs with higher work engagement can control and regulate their emotions, found that about 70% of psychiatric nurses had low work engagement. Bechtoldt et al. (2011) suggested that the level of work engagement in workers engaged in emotional labor is related to awareness of emotions. Thus, it may be that evaluating the EI of the MHNs that participated in this study had an effect on their sense of fulfillment in their work.

Self-awareness is not a skill that can be improved by the individual. That is because reflection is elicited and developed by involvement with and support for others (Smith and Gray, 2001; Clouder and Sellars, 2004). Therefore, involvement with others is needed in the reflective practice and thought of nurses. Ishii (2018) suggested three things to raise the EI of MHNs: education programs for newly graduated nurses, planning and methods of postgraduate education programs, and evaluation of education programs. However, EI will probably not improve with new nurse training and continuous learning within hospitals alone. MHNs in Japan need to actively participate in workshops and conferences outside their own workplace, be involved with nurses and many specialists from other institutions, learn, and reflect back on themselves. Training for learners to reflect back on their own emotions through dialog with supervisors or records during or after practice in basic nursing education is also an issue.

### Clinical Implications

Mental health nurses always faces the different emotions of patients with mental disorders and their families. If they are a profession, they will believe it is natural. However, MHNs’ own EI rating was low. The reason may be the emotion regulation of MHNs as a profession. It is important for MHNs to remove the strong frames (such as ‘must…’ and ‘should be…’) in the profession. Therefore, MHNs need a working environment and team emotional engagement that can communicate not only positive but also negative emotions.

### Limitations

The participants in this study were limited to five hospitals in the Kyushu region of Japan, and generalization may not be possible. Moreover, there is a possibility that participants recalled and evaluated their own families or friends, in addition to their patients, for “others” when responding to OEA items on the J-WLEIS. Thus, it is difficult to derive firm conclusion based on the results of this study, and interpretation needs to be done carefully.

In the future, the universality of results will be increased by conducting surveys on a more national scale. It will also be necessary to elucidate actual involvement with others to raise self-awareness in the reflective practices of MHNs.

## Conclusion

The purpose of this study was to elucidate the characteristics of EI in MHNs. The analysis results indicated that the self-awareness of MHNs was significantly lower than that of nurses in other fields. The results of this study provide a basis for future research on the EI of nurses in Japan. However, further review and investigation will be needed regarding nurses’ assessment of their own emotions and the emotions of others, and it will be important to develop an EI scale based on the characteristics of Japanese nurses. The kinds of people nurses are involved with and the content of their support for others also needs to be clarified.

## Data Availability

All datasets generated for this study are included in the manuscript and or the supplementary files.

## Ethics Statement

The purpose of the study was explained to all the participants during the delivery of the anonymous self-reported questionnaire. A returned questionnaire was considered to be indicative of one’s consent to participate. This study was approved by the Ethics Committee of Saga University, Saga, Japan (Approval no. 25-6).

## Author Contributions

SI searched and reviewed the study, performed the data collection and analyzed the data, and wrote the manuscript. EH critically reviewed the manuscript and supervised the entire research process. Both authors contributed to data interpretation and writing the manuscript and approved the final version of the manuscript for submission.

## Conflict of Interest Statement

The authors declare that the research was conducted in the absence of any commercial or financial relationships that could be construed as a potential conflict of interest.
